# Availability and use of public access defibrillators in Busan Metropolitan City, South Korea

**DOI:** 10.1186/s40064-016-3201-6

**Published:** 2016-09-09

**Authors:** Chang Guk Yoon, Jinwoo Jeong, In Ho Kwon, Jae Hoon Lee

**Affiliations:** 1 Department of Emergency Medicine, Dong-A University Hospital, Busan, 49201 Republic of Korea; 2 Department of Emergency Medicine, College of Medicine, Dong-A University, Busan, 49201 Republic of Korea

**Keywords:** Cardiac arrest, Public access defibrillation, Automated external defibrillators, Cardiopulmonary resuscitation

## Abstract

Out-of-hospital cardiac arrest (OHCA) is considered an important health issue worldwide, and early defibrillation is a key element for a favourable prognosis. In South Korea, public access defibrillation (PAD) programmes were initiated in 2007. However, the impact of PAD programmes on OHCA survival rates remains unclear. This study evaluated the deployment and maintenance status of public automatic external defibrillators (AED), including how frequently they were used, in Busan Metropolitan City, South Korea. Managers of possible AED sites were first contacted by telephone and asked to confirm the possession of an AED. AED suppliers were contacted for AED sales records to identify missing AED sites. AEDs located in ambulances and medical institutions were not included. Investigators visited confirmed AED sites and completed a checklist on AED maintenance and use. In total, 206 AEDs were located, indicative of an AED density of 0.268 AED/km^2^ and a prevalence of 6.07 per 100,000 in Busan Metropolitan City. We found that public AEDs had been used for resuscitation only 15 times, an average rate of use of once every 26.3 years. Our results indicate that AEDs in Busan Metropolitan City are underused according to the guidelines, and several are in low-priority locations. We believe that AED deployment based on cardiac arrest statistics is important to optimise layperson AED training and utilisation.

## Background

Out-of-hospital cardiac arrest (OHCA) is considered an important health issue worldwide (Agerskov et al. 2015). The OHCA survival rate is low, and early defibrillation is a key element for a favourable prognosis (Perkins et al. 2015; Priori et al. 2004). Several studies have suggested that public-access defibrillation (PAD) programmes, which train laypersons in applying automatic external defibrillators (AEDs) to victims prior to arrival of emergency medical service (EMS) providers, increase OHCA survival rates (Kitamura et al. 2010; Hazinski et al. 2005; Ringh et al. 2015). In South Korea, PAD programmes were initiated in 2007, when the EMS Act mandated that laypersons in certain places be able to use AEDs. However, the impact of PAD programmes on OHCA survival rates remains unclear. In addition, reports have indicated underutilisation of AEDs. A study from Ansan City, Korea, revealed no reported case of a layperson using an AED during the 4-year study period (Cho et al. 2014). Based on this finding, this study evaluated the deployment and maintenance status of public AEDs, including how frequently they were used, in Busan Metropolitan City, South Korea.

## Methods

Between 1 October 2013 and 31 December 2013, data were collected as part of an administrative project by the Busan Metropolitan City government. In 2013, Busan City included an area of 770 km^2^ and had a residential population of approximately 3,393,191.

Managers of possible AED sites were first contacted by telephone and asked to confirm possession of an AED. Potential AED sites included airports, railway stations, ferry and bus terminals, casinos, correction facilities, governmental offices, sports facilities (e.g., stadiums, racetracks and velodromes), apartments with more than 500 households, public telephones, registered non-profit organisations, schools and other educational facilities. AED suppliers were contacted for AED sales’ records to identify missing AED sites. AEDs located in ambulances and medical institutions were not included. Investigators visited confirmed AED sites and completed a checklist on AED maintenance and use. The following information was collected via checklist: name and type of facility, locations of AEDs in the facility, license of the individual overseeing the AEDs, periods of inspection, expiration date of the pads, AED operable condition and the number of times the AED had been used for resuscitation.

Potential first responders to AED sites were invited and provided with 4 h of CPR and AED training after collecting AED maintenance data. They were asked to complete a survey questionnaire regarding their previous confidence in CPR and AED use.

MedCalc software (ver. 15.6; MedCalc Software, Mariakerke, Belgium) was used for statistical analyses. A *p* value <0.05 indicated statistical significance.

## Results

In total, 206 AEDs were located, indicating an AED density of 0.268 AED/km^2^ and a prevalence of 6.07 per 100,000 population in Busan Metropolitan City. The maintenance status of these AEDs is summarised in Table 1. Of the AEDs, 196 had a reported installation date and were included for further analysis. We found that public AEDs had been used for resuscitation only 15 times, an average rate of use of once every 26.3 years (Table 2). A non-profit organisation, the ‘Korea Injury Prevention Association’, had used three different AEDs a total of seven times. Correction facilities had used two different AEDs a total of three times. Other organisations had used an AED either once or never at the time of this study. Sites at which physicians oversaw AEDs were more likely to report AED use (*p* < 0.001). In total, 85 AEDs (41.3 %) were located in closed spaces such as offices, limiting access to designated first responders, while the other 121 (58.7 %) were located in open spaces allowing access by laypersons. AEDs owned by apartment buildings tended to be located in closed spaces, while those in transport terminals were located in open spaces. AED use was not influenced by whether the AED was located in an open or a closed space. Among the various sites, other maintenance status parameters did not differ significantly.

**Table 1:** Maintenance status of automatic external defibrillators in Busan Metropolitan City, South Korea

Facility type	Inspected monthly	Pad life	Machine operable	Stored in secure location	Stored in an open location	Total (%)
Apartment	37 (71.2)	52 (100.0)	52 (100.0)	50 (96.2)	4 (7.7)	52 (100.0)
Commercial facility	12 (66.7)	18 (100.0)	18 (100.0)	18 (100.0)	8 (44.4)	18 (100.0)
Education organisation	13 (92.9)	14 (100.0)	14 (100.0)	12 (85.7)	7 (50.0)	14 (100.0)
Government office	11 (100.0)	11 (100.0)	11 (100.0)	11 (100.0)	10 (90.9)	11 (100.0)
Ship	5 (100.0)	5 (100.0)	5 (100.0)	5 (100.0)	1 (20.0)	5 (100.0)
Non-profit organisation	10 (83.3)	12 (100.0)	12 (100.0)	12 (100.0)	4 (33.3)	12 (100.0)
Sports stadium	8 (100.0)	7 (87.5)	7 (87.5)	8 (100.0)	3 (37.5)	8 (100.0)
Telephone booth	50 (90.9)	55 (100.0)	55 (100.0)	54 (98.2)	55 (100.0)	55 (100.0)
Transportation terminal	26 (100.0)	26 (100.0)	26 (100.0)	26 (100.0)	26 (100.0)	26 (100.0)
Others	5 (100.0)	4 (80.0)	5 (100.0)	4 (80.0)	3 (60.0)	5 (100.0)
Total	177 (85.9)	204 (99.0)	205 (99.5)	200 (97.1)	121 (58.7)	206 (100.0)

**Table 2:** Utilisation of public automatic external defibrillators compared to last time of deployment

Facility type	Number of AEDs	Months since installation^a^	AED-year	AED use in actual cases	AED-year per actual case
Apartment	47	3.9 (2.3–12.8)	28.1	1	28.2
Commercial facility	18	11.2 (5.6–53.2)	38.4	0	N/A
Education organisation	14	46.0 (7.3–75.3)	55.1	1	55.1
Government office	10	30.9 (21.1–60.4)	30.0	1	30.0
Ship	5	38.1 (30.8–38.3)	13.5	0	N/A
Non-profit organisation	8	31.8 (12.2–48.1)	20.8	7	3.0
Correction facility	2	65.3 (60.0–70.8)	130.7	3	3.6
Sports stadium	8	53.5 (36.0–55.4)	30.8	0	N/A
Telephone booth	55	17.8 (7.3–20.1)	67.6	0	N/A
Transportation terminal	26	36.5 (36.5–52.5)	91.4	0	N/A
Others	3	22.3 (22.3–40.2)	7.6	1	3.8
Total	196	19.0 (5.6–36.5)	394.2	15	26.3

*AED* automatic external defibrillator

^a^Presented as median (interquartile ranges)

In total, 125 potential responders attended the CPR and AED training. Their degree of confidence with CPR and AED before initiating the training course is summarised in Table 3. Before participating in the course, only 39.1 % replied that they could provide CPR to those without consciousness and breathing.

**Table 3:** Confidence of potential responders in providing cardiopulmonary resuscitation and using an automatic external defibrillator before participating in the training course

Facility type	Number of participants	Can provide CPR (%)	Can use an AED (%)
Apartment	48	18 (37.5)	23 (47.9)
Commercial facility	8	7 (87.5)	6 (75.0)
Educational organisation	6	4 (66.7)	4 (66.7)
Government office	1	0 (0.0)	0 (0.0)
Non-profit organisation	7	2 (28.6)	4 (57.1)
Sports stadium	7	4 (57.1)	3 (42.9)
Telephone booth	37	7 (18.9)	17 (45.9)
Transportation terminal	8	6 (75.0)	6 (75.0)
Others	3	0 (0.0)	1 (33.3)
Total	125	48 (38.4)	64 (51.2)

*CPR* cardiopulmonary resuscitation, *AED* automatic external defibrillator

## Discussion

We found a total of 206 AEDs with layperson access, which were well maintained, with 99.5 % in operable condition. However, use of an AED in an arrest situation was rare, with an average of once per 26.3 years. Only about half of the potential responders had confidence in the use of an AED.

Based on population and area, there are fewer public AEDs in Busan Metropolitan City than in cities in developed countries (Table 4). However, it is important to note that a substantial part of the area included in Busan City is uninhabited mountains (Chan et al. 2013; Deakin et al. 2014; Ho et al. 2014; Hansen et al. 2014). Despite this, our results showing a relatively low number of AEDs in Busan City may be attributed to the more recent introduction of public AEDs in Korea. It was not until 2007 that installation of AEDs became mandatory in selected public places and layperson’s use of AEDs became legal (Bae 2008).

**Table 4:** Number of automatic external defibrillators per population and area in various cities

Area (year)	Number of AEDs	Population (×10^6^)	AED/100,000 population	Area (km^2^)	Density AED/km^2^
Busan Metropolitan City (2013)	206	3.4	6.1	770	0.3
New Territories, Western Region of Hong Kong (2013) (Ho et al. 2014)	207	1.1	19.4	223	0.9
Copenhagen (2011) (Hansen et al. 2014)	552	0.6	92.0	97	5.7
Toronto (2009) (Chan et al. 2013)	1669	2.5	66.8	630	2.6

*AED* automatic external defibrillator

Although the Korean government is attempting to increase AED density, the number of AEDs does not guarantee successful use or increased OHCA survival rates (Atkins 2010). Our study estimated that on average, an AED is used only once every 26 years. This is in contrast with the European Resuscitation Council (ERC) and American Heart Association (AHA), who recommend that AEDs be placed at sites where one cardiac arrest is expected every 2–5 years (Aufderheide et al. 2006; Perkins et al. 2015). The low rate of AED use raises concerns regarding the benefits and cost-effectiveness of public AEDs. Previously published studies have suggested that there are limitations associated with PAD programmes, including their unguided and unplanned nature (Atkins 2010; Folke et al. 2009; Haskell et al. 2009). Nishi et al. (2015) reported, based on EMS data, that a large percentage of prehospital AED application was by healthcare providers in care facilities. Their findings correspond with our result that AEDs are likely to be used when physicians are in charge of AEDs, although AEDs in care facilities were not considered PADs in our study, similar to previous community-based studies (Folke et al. 2009; Hansen et al. 2013; Ho et al. 2014).

For a successful PAD programme, several elements are necessary: planned responses, training anticipated rescuers, association with local EMS systems and continued quality improvement (e.g., on-site AED maintenance) (Aufderheide et al. 2006).

Planned response systems include optimised AED deployment where the risk of sudden cardiac arrest is highest. Several studies have addressed the disparity between OHCA high-risk locations and AED deployment (Deakin et al. 2014; Hansen et al. 2014; Folke et al. 2009; Moon et al. 2015; Agerskov et al. 2015). Some studies have attempted to identify optimal AED locations by using registries of historical cardiac arrest data and mathematical analyses to prioritise cost-effective locations (Chan et al. 2013; Folke et al. 2009; Moran et al. 2015). According to these studies, apartments and unattended telephone booths are considered low-priority areas and yet they had 49.5 % of all public AEDs identified in this study (Atkins 2010; Moran et al. 2015; Hansen et al. 2013). Community fundraisers and private foundations may have influenced legislature to place AEDs in low-risk areas, such as schools (Atkins 2010). In Korea, the EMS Act mandates that apartment complexes larger than 500 households install AEDs, which has not been supported by the results of previous studies.

Instead of requiring a large number of potential rescuers to be trained, PAD programmes that train only anticipated responders and that have an on-site action plan are more likely to be successful (Atkins 2010; Aufderheide et al. 2006). Our study revealed that AEDs in the possession of trained individuals and healthcare professionals (e.g., doctors in correction facilities and non-profit organisations providing emergency support during a mass gathering) were more likely to be used than AEDs located in random locations (e.g., telephone booths). Measuring the training level of the general population is impractical because there are various sources of formal or informal training (Brooks et al. 2015). A face-to-face survey conducted in the UK revealed that only 2.1 % of the general public would attempt to use a PAD when faced with someone in cardiac arrest, while 79 % of interviewees had some confidence in their CPR skills, indicating that only a small percentage of the people with knowledge of BLS skills are willing to provide BLS to strangers (Brooks et al. 2015). We believe that the training of designated AED responders should take precedence over the training of random volunteers (Koster 2005). The EMS Act in Korea recognises some groups of citizens as potential first responders and the municipal government of Busan provides first aid and CPR training to 20,000–30,000 potential responders every year. Legally recognised potential first responders include ambulance drivers, bus drivers, police officers, flight and train attendants, sports trainers and tour guides. A large percentage of possible responders in PAD locations have been outside the reach of the CPR training provided by the government, which may explain the low confidence in CPR found in this study. The Busan Metropolitan City government has been operating a CPR training programme for possible responders in PAD locations since 2013, following the data collection period of this study. The effect of that education programme should be reviewed in future studies.

It is important to note that our study has some limitations. For example, we anticipate that not all AEDs were accounted for, in accordance with other previously published studies that focused on public AEDs (Ringh et al. 2015; Folke et al. 2009; Ho et al. 2014). The self-reported confidence in CPR and AED use should be interpreted with caution because discrepancies between self-confidence and actual knowledge have been reported (Brooks et al. 2015), and there may be bias from the group attending the training course.

## Conclusions

Our results indicate that AEDs in Busan Metropolitan City are underused according to the recommendations published by the ERC and AHA, and several are in low-priority locations. We believe that AED deployment based on cardiac arrest statistics will be necessary to optimise layperson AED training and use.
